# The Effects of Garlic and Red Chilli Pepper Powder on Physicochemical, Microbiological, and Sensory Properties of Cincalok

**DOI:** 10.1155/2021/2882005

**Published:** 2021-10-12

**Authors:** Risa Nofiani, Novi Maulidi Syahmurdiandi, Puji Ardiningsih

**Affiliations:** Department of Chemistry, Faculty of Mathematics and Natural Sciences, Universitas Tanjungpura, Jl. Prof. Dr. Hadari Nawawi, Pontianak 78124, Indonesia

## Abstract

Cincalok, a traditional fermented shrimp, is prepared by mixing rebon shrimps (*Acetes* sp) with coarse salt and granulated sugar in a certain ratio. This research was aimed at studying the effect of adding garlic and red chilli pepper powder on the physicochemical, microbiological, and sensory properties of cincalok. Cincalok was made to be three recipes, namely, original cincalok, A, consists of 2 kg of rebon shrimp, 400 g of granulated sugar, and 100 g of coarse salt; B (A ingredients plus 20 g of red chilli pepper powder); and C (A ingredients plus 20 g of garlic powder). Sensory analysis was conducted on recipe A, and the colour was observed by the naked eye on days 0, 2, 4, 6, 8, 13, 18, 23, 28, 33, 43, 60, 90, 120, 150, and 180. According to the highest criterion score on sensory results, the panellists chose day 6 as the best fermentation for recipe A. The colour of recipe A started changing from pink to a light brown colour on the surface on day 28. Therefore, the physicochemical, microbiological, and sensory properties of each recipe were analyzed for 28 days. Overall, the water, ash, and fat content; titratable acid (TA); total volatile base nitrogen (TVBN); and amino acid nitrogen (AAN) showed insignificant differences (*p* > 0.05) among the recipes during 28 days of the same observation. The crude protein, pH, and free fatty acid (FFA) of recipe C were significantly different (*p* < 0.05) from recipes A and B. All recipes contained the total count of mesophilic anaerobic bacteria (TMABs) and the lactic acid bacteria (LABs) except *Bacillus cereus*, *Clostridium perfringens*, *Staphylococcus aureus*, and *Enterobacteriaceae* for all observation times. The highest criterion score for consumer acceptability was awarded for recipe C followed by recipes B and A. The addition of garlic and red chilli pepper powder affected the physicochemical, microbiological, and sensory properties of cincalok.

## 1. Introduction

Cincalok is a small shrimp fermentation production that is known in South Asia with various names, i.e., cincalok or kecaluk or ronto from Indonesia, cincalok from Malaysia, and balao-balao from the Philippines [1–4]. Cincalok is generally made from fresh and tiny shrimps, coarse salt, and other ingredients (cooked rice or sugar or palm sugar or tapioca flour) and naturally fermented for 3-14 days depending on region or country [1–7]. Cincalok colour is usually light pink, but some producers add red food colouring to cincalok to obtain dark pink cincalok. Cincalok has a specific flavour and savoury taste resulting from protein degradation by microorganisms, producing short-chain peptides or free amino acids. Cincalok can be categorized as salty and sweet depending on its taste, which differs based on salt concentration in the cincalok recipe. Cincalok is usually stewed with shallot, garlic, and chilli, but it also can be consumed without cooking together with rice.

Chilli pepper and garlic are seasonings to develop food aroma, flavour, taste, and appearance. Chilli pepper and garlic are also widely used in fish fermentation and can contribute to increasing the safety of products [8]. Red chilli pepper (*Capsicum annuum* L. var. Longum Sendt) is rich in vitamins, minerals, and capsaicin [9]. Capsaicin originates from the spicy taste of the food [10]. Besides, red chilli pepper can be used to shorten the fermentation time and inhibit various pathogenic microorganisms. For example, adding spices can decrease bacteria in fish sauce fermentation [11]. Garlic (*Allium sativum* L) contains allicin that plays a role in antimicrobial activities [12, 13]. Som-pak, a Thai low-salt fermented fish product, is enriched with various garlic concentrations (Bernbom et al. [14, 15]). The som-pak added with 8% garlic can decrease pH to 4.5 in 2 days [16]. The Korean salted and fermented anchovy product, Myeolchi-jeot, adds garlic extract to inhibit biogenic amine production *in vitro* and *vivo* [17].

In this study, the original cincalok (recipe A) was supplemented with red chilli pepper powder (recipe B) and garlic powder (recipe C) and fermented. Physicochemical, microbiological, and sensory properties of cincalok recipes A, B, and C were analyzed. This study was aimed at investigating the effect of adding garlic and red chilli powder in each recipe on the physicochemical, microbiological, and sensory properties of cincalok.

## 2. Materials and Methods

### 2.1. Preparation of Cincalok

Rebon shrimps were bought from a fisherman in Sungai Duri, Bengkayag Regency, West Kalimantan, Indonesia, then directly made cincalok at a cincalok producer in Sungai Duri, Bengkayang, West Kalimantan. Cincalok was prepared using three recipes, namely, A (2 kg of rebon shrimp; 400 g of granulated sugar, no brand; and 100 g of coarse salt, Bentul brand), B (recipe A plus 20 g of red chilli pepper powder, Koepoe koepoe brand), and C (recipe A plus 20 g of garlic powder, Koepoe koepoe brand). Fresh rebon shrimps (small shrimp) were sorted out from the impurity (i.e., small fish) then rinsed with seawater. The fresh and cleaned rebon shrimps were carefully mixed with all of the ingredients following the recipes and put in a glass container, covered with a lid, and then incubated at room temperature. Sensory evaluation of each recipe was carried out in Sungai Duri, Bengkayag Regency, West Kalimantan, Indonesia. At the same time, the physicochemical and microbiological analyses were conducted in the Biotechnology Laboratory, Faculty of Mathematics and Natural Sciences, Universitas Tanjungpura, Indonesia.

In this study, we divided two steps in cincalok production. In the first step, the sensory and colour were evaluated in recipe A using the naked eye on days 0, 2, 4, 6, 8, 13,18, 23, 28, 33, 43, 60, 90,120, 150, and 180. These results were used to determine the duration of physicochemical, microbiological, and sensory analysis for recipes A, B, and C.

### 2.2. Physicochemical Analysis

Each sample was homogenized using a hand blender before physicochemical analysis.

#### 2.2.1. Proximate Analysis

The proximate analysis (moisture, ash, crude protein, and fat) in this study was carried out based on the procedures described in the AOAC official method [18] and Indonesian National Standard (INS) number 01-2891-1992 [19]. The moisture was carried out using an oven. The ash content was conducted using the gravimetric technique. The crude protein content was conducted using the semi-micro-Kjeldahl and calculated using 6.25 as the protein factor. The fat was analyzed using a Soxhlet extractor with n-hexane as solvent.

#### 2.2.2. pH

Two g of homogenate was diluted with 18 mL of distilled water. The mixture was measured using a pH-meter digital (EUTECH, CyberScan 300).

#### 2.2.3. Free Fatty Acid (FFA)

The FFA was analyzed using acid-base titration [20]. A 28.2 g of the homogenate was added with 50 mL of warm and neutral ethanol, and two drops of phenolphthalein were then titrated with NaOH 0.1 N until light pink. The FFA was expressed as oleic acid. The FFA was calculated as follows:

(1) $$\begin{array}{c} \%\text{FFA}=\frac{\text{mL NaOH}\times \text{N NaOH}\times 282}{\text{sample weight}\times 1000}\times 100. \end{array}$$

#### 2.2.4. Titratable Acidity (TA)

The TA was determined by acid-base titration according to the AOAC official method 942.15 [18]. One g of the homogenate was diluted with 9 mL of distilled water and centrifuged at 3.077 × g for 2 hours, and then, the supernatant was collected. Five mL of the supernatant was diluted with distilled water until 10 mL and boiled for a minute. After cooling down, the supernatant was added 2-3 drops of phenolphthalein and titrated using 0.1 M sodium hydroxide until a light pinky solution formed. The TA was calculated as lactic acid with the formula as follows [21]:

(2) $$\begin{array}{c} \%\text{TA }\left(\text{mM}/\text{mL}\right)=\frac{\text{M NaOH}\times \text{V NaOH}\times 0.9}{\text{sample volume}}\times 100\%. \end{array}$$

#### 2.2.5. Total Volatile Base Nitrogen (TVBN)

The TVBN was determined using steam distillation described by the Codex Alimentarius Committee (1968) [22]. Twenty-five g of homogenate sample was extracted using 50 mL of trichloroacetic acid 7.5% and then centrifuged at 1.924 × g for 10 min to obtain supernatant. The supernatant was filtered using a filter Whatman No. 1. 6 mL of NaOH 10% was added to 25 mL of the filtrate, and distillation was carried out. The distillate was collected into 10 mL of boric acid 4% and two drops of indicator (methyl red and bromocreosol green). The collection was stopped when the solution changed from red to green. Finally, the solution was titrated with sulfuric acid 0.1 N using a 0.01 mL graduated microburette until the colour turned green to red. The TVBN was calculated as follows:

(3) $$\begin{array}{c} \text{TVBN }\left(\text{mg}\frac{\text{N}}{100\text{ g}}\right)=\frac{\text{V }\left({\text{H}}_{2}{\text{SO}}_{4}\right)\times 16.8\text{ mg nitrogen}}{100\text{ g of sample}}. \end{array}$$

#### 2.2.6. Amino Acid Nitrogen (AAN)

The AAN was determined by formol titration [23]. A two mL sample of homogenate was added to 18 mL of distilled water, and the pH was adjusted to 8.5 using NaOH 0.1 M. To the sample, 5 mL of formaldehyde 37% was added. After 2 min, the sample was titrated using NaOH 0.1 M until constant pH 8.5 (stable 30 s). AAN was calculated as follows:

(4) $$\begin{array}{c} \text{AAN }\left(\text{mM}/\text{g}\right)=\frac{\text{M NaOH}\times \text{V NaOH}}{\text{sample weight}}. \end{array}$$

### 2.3. Microbiological Analysis

Microbiological analysis was conducted following suspension of A 5 g of sample in 45 mL of sterilized saline buffer (0.9% of NaCl) to obtain a suspension. 50 *μ*L of the suspension sample (dilution 10^−1^) was inoculated on a specific media and incubated at 30°C for analysis of the total count of mesophilic anaerobic bacteria (TMABs), total count of halotolerant bacteria 10 (THBs 10), THB 17.5, lactic acid bacteria (LABs), fungi, *Bacillus cereus*, *Clostridium perfringens*, *Enterobacteriaceae*, and *Staphylococcus aureus*. The TMABs, THBs 10, and THB 17.5 were determined using PCA media enriched with 15 g/L, 10 g/L NaCl, and 17.5 g/L of NaCl, respectively [24]. Each bacterial colony on the surface media was counted as a log colony-forming unit (CFU) after incubation of 2-3 days.

For LAB count, MRS media enriched with 1% of CaCO_3_ and anaerobically incubated was used [25]. A clear zone around colonies observed on days 3-4 was counted as the number of LABs.

Determination of fungal total was carried out on *Oxytetracycline Glucose-Yeast Extract Agar* (OGYE) media supplemented with NaCl 5%, streptomycin sulfate 0.25 mg/mL and penicillin G 0.25 mg/mL [24]. The fungal colony was counted on day 5.

The sample was inoculated on *Mannitol Egg Yolk Polymyxin* (MYP) supplemented with 5% NaCl and incubated on days 2-3 to determine *B. cereus* [24]. The pink colonies surrounded by zone precipitation were counted and followed further identification based on *Bergey's Manual of Determinative Bacteriology* [26].

*S. aureus* was determined using *Mannitol Salt Agar* (MSA) supplemented with 5% NaCl. The yellow colonies with yellow zones were tested for coagulase; then, the positive coagulase test was counted as log CFU of *S*. *aureus*.

*Enterobacteriaceae* was evaluated following a procedure described by and Han et al. and Viji et al. using a selective medium, *Violet Red Bile Glucose Agar* (VRBG), supplemented with NaCl 5% [24, 27]. The large colonies with purple haloes were counted as Enterobacteriaceae.

*Tryptose Sulfite Cycloserin Agar* (TSCA) supplemented with NaCl 5% was used to count the total of *C. perfringens*([24, 28]; Tirillini et al. 2019). After anaerobic incubation at 37°C for 18-24 h, black colonies were counted as log CFU followed by the confirmation tests, namely, fermentation of lactose, gelatin liquefaction, motility, and nitrate reduction. If the black colony was *C. perfringens*, the fermentation of lactose and gelatin liquefaction showed a positive result, and the motility and nitrate reduction showed a negative result.

### 2.4. Sensory Analysis

The sensory analysis was conducted using the hedonic method according to INS number 2346:2015 [29]. The consumer acceptability was measured using a nine-point hedonic rating scale (1, dislike extremely; 2, dislike very much; 3, dislike moderately; 4, dislike slightly; 5, neither like nor dislike; 6, like slightly; 7, like moderately; 8, like very much; and 9, like extremely). Each sample was prepared in a small container with a spoon and drinking water and served to each semitrained panellist. Each panellist was instructed to cleanse their palate using the drinking water before each testing. Evaluation of each sample by panellists was carried out in a big room to evaluate the consumer acceptability of each recipe. The panellists chosen for this study belong to a community that consumes cincalok and are cincalok's producers. Consumer acceptability for each recipe was analyzed using the analytical hierarchy process (AHP) technique with four criteria: taste (sweet, salt, sour, and yummy), aroma, texture, and product appearance. The consistency level of the panellist was calculated by consistency ratio (CR) as follows [30]:

(5) $$\begin{array}{c} \text{CR}=\frac{\text{CI}}{\text{RI}}, \end{array}$$

where CI is consistency index and RI is the average value of CI.

### 2.5. Statistical Analysis

The physicochemical analysis of this experiment was conducted using a completely randomized design and repeated three times. The variation among the treatments was analyzed through one-way analysis of variance (ANOVA) using Tukey's test as the post hoc comparison test (*p* < 0.05).

## 3. Results and Discussion

### 3.1. Change in Physical Cincalok during the Fermentation

**Figure 1:**
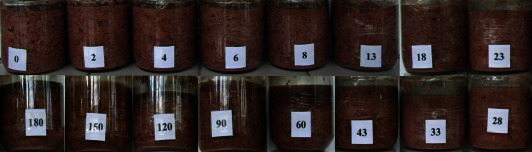
Observation of recipe A for 180 days of fermentation. The number showed the fermentation time, day.

Change in physical cincalok during the fermentation was carried out in 2 steps. In the first step, recipe A was observed for its colour and texture by the naked eye for six months of fermentation (days 0, 2, 4, 6, 8, 13,18, 23, 28, 33, 43, 60, 90, 120, 150, and 180). Recipe A showed a light pink colour on day 0. The light brown colour on the cincalok surface appeared on the 28^th^ day and gradually changed to brown on the 33^rd^ day (Figure 1). The brown colour was caused by a browning reaction. Therefore, recipes A, B, and C were analyzed for the physicochemical, microbiological, and sensory properties for 28 days.

**Figure 2:**
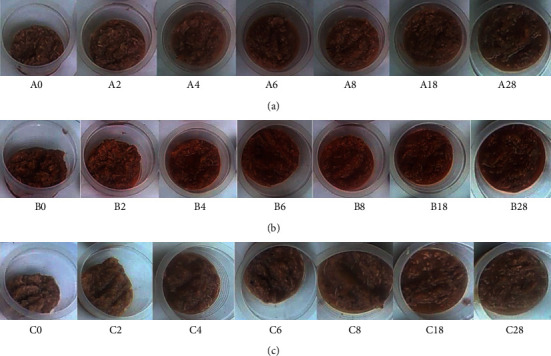
The appearance of cincalok: (a). recipe A; (b). recipe B; (c). recipe C. The number showed the fermentation time (day).

In the second step, the researchers further observed the colour and texture for recipes A, B, and C on days 0, 2, 4, 6, 8, 18, and 28. Recipes A, B, and C exhibited different pink colours. Recipe A was slightly brighter pink than recipe B, which showed a dark pink colour caused by the addition of red chilli pepper powder (Figure 2). The addition of garlic powder caused a pale pink colour on recipe C. During the observation, the colour of each recipe gradually changed from pink and became brighter pink from day 4 to day 8. On day 18, the colour became darker (light brown) due to the browning reaction, and the texture became slightly mushy since the protein degradation. This brown intensity and shrimp degradation continually increased until the end of the observation. The browning reaction is also reported on the other fermentation products such as soy sauce and fish sauce [31]. This colour change probably affected consumers' acceptance to consume each recipe. Physical changes, i.e., texture and colour, are one of the parameters to determine the expired date of the product [32]. Therefore, in recipes A, B, and C, the expiration date before the 18^th^ day can be proposed based on the colour and product appearance. However, the addition of garlic and red chilli pepper powder affected the product appearance of cincalok.

The commercial cincalok from West Kalimantan is usually ripe after fermentation for 3 to 6 days [7]. Recipes A, B, and C showed a brighter pink color from day 4 to day 8, which was probably a sign of the cincalok ripeness. All of the cincalok products usually show different pink colours caused by different recipes and fermentation times. However, no brown colour is detected in all of the commercial cincalok products in the market.

### 3.2. Change in Proximate Compositions of Cincalok during the Fermentation

The proximate compositions of recipes A, B, and C were determined (moisture, ash, fat, and crude protein) on days 0, 2, 4, 6, 8, 18, and 28 (Table 1). The proximate compositions among the recipes on the same incubation time almost showed an insignificant difference (*p* > 0.05) (Table 1). The proximate compositions were almost significantly different (*p* < 0.05) in the same recipe with different incubation times except for the fat content of recipes A, B, and C and the moisture of recipe B (Table 1). The addition of red chilli pepper (recipe B) and garlic powder (recipe C) on recipe A (original cincalok) generally insignificantly changed (*p* > 0.05) the proximate compositions.

The moisture on day 0 was around 54-55% for all recipes (Table 1). The moisture profile showed a significantly increased (*p* < 0.05) fermentation time for recipes A and C but not recipe B. The final moisture among the recipes was around 60-61%. It is lower than the commercial cincalok from Indonesia, approximately 67.6% [5, 7], and cincalok from Malaysia [6].

Ash content indicates minerals and inorganic content in food. The ash content of each recipe during the fermentation showed a fluctuation pattern, mainly for recipe C (Table 1). Besides, the ash content of recipes A and B increased from day 0 to day 16 then decreased after that. The fluctuation in the ash content during the fermentation also is reported on fermented pitaya stem flour [33]. The ash content with different incubation times for recipe A showed significant differences (*p* < 0.05) toward recipes A and C. Microorganism activities during the fermentation could cause fluctuation in the ash content. For example, LAB-fermented sorghum flour and LAB-fermented maize flour significantly increase (*p* < 0.05) than naturally fermented sorghum flour [34].

The fat content of rebon shrimp as a raw material was low, approximately 0.6-1.9% (Table 1). The fat content fluctuated among the recipes ranging from 0.5 to 2.5% during the fermentation (Table 1). However, this change showed an insignificant difference (*p* < 0.05) in each recipe with different fermentation times or with the same fermentation time.

The crude protein trend tended to decrease for all recipes during the observations (Table 1). The protein during fermentation was probably changed into ammonia by microorganism activities, causing a decrease in the protein content [35]. A decrease in protein content is also reported by Pranoto et al. on sorghum flour fermented by *Lactobacillus plantarum* [36]. The crude protein among recipes A, B, and C showed an insignificant decrease (*p* > 0.05) during the observation.

### 3.3. Change in Physicochemical Cincalok during the Fermentation

The acidity affects food flavour, colour, taste, and preservation, which is an important parameter for assessing product quality [37]. The TA is better to assess the flavour and taste of food than the pH [21]. The TA measures the total organic acid concentration contained within a food. The pH value measures proton concentration.

The pH of all the recipes for certain fermentation times significantly decreased from 8 to 5, but there are insignificant differences among recipes for the particular observation time (Table 2). The recipe pH decrease was caused by microorganisms producing organic acids (i.e., lactic, tartaric, malic, and citric), for example, the LABs producing lactic acid. On day 6, the LABs were not present on recipes A and B while they were detected on recipe C (Table 3). However, the pH of recipes A and B was significantly lower (*p* < 0.05) than that of recipe C. Lower pH of recipes A and B than recipe C might be caused by microorganisms other than LABs.

The TA showed an insignificant difference (*p* > 0.05) among recipes A, B, and C but significantly different from each recipe during the fermentation time (Table 2). The addition of red chilli pepper (recipe B) and garlic powder (recipe C) did not affect the TA value compared to recipe A.

Protein degradation during fermentation is caused by the activities of proteolytic bacteria and proteolytic enzymes that can be measured from the TVBN and FAN values. The TVBN measures the total amount of ammonia and amines (dimethylamine and trimethylamine). The TVBN value can be used to assess food quality, particularly seafood spoilage. The TVBN also is used to measure the ripeness level of fermentation [38]. The TVBN profile of recipes A and C increased drastically for the first 18 days and decreased for day 28 (Table 2). The addition of red chilli pepper or garlic powder on cincalok did not significantly affect (*p* > 0.05) the TVBN value among the recipes.

Besides the TVBN, the AAN content also indicates the proteolytic activity during fermentation [13]. The AAN is used to measure the concentration of small molecular weight nitrogenous compounds (amino acids, small peptides, and ammonium ions). The AAN is vital to stimulate yeast growth. The AAN of each recipe showed a significant increase (*p* < 0.05) during the fermentation of each recipe event though insignificant differences among the recipes were observed (Table 2).

FFA is produced from the hydrolysis of lipid by microorganisms or lipase enzymes of raw materials. Further enzymatic or nonenzymatic oxidation of the FFA produces volatile and nonvolatile undesirable flavour compounds that affect the food quality, particularly flavour, colour, nutrition, and texture [39]. The FFA content of each cincalok recipe significantly increased (*p* < 0.05) during the fermentation period, while the cincalok recipe for the same fermentation time generally showed an insignificant difference (*p* < 0.05) (Table 2). The garlic and red chilli pepper powder added into cincalok did not inhibit lipid hydrolysis during the cincalok fermentation.

### 3.4. Change in Microbiological Cincalok

The microbiological profile of products is a fundamental step for assessing the safety and quality of the food products, particularly the ones based on a spontaneous fermentation such as cincalok. The microbiological profile also affects the sensory, textural, and nutritional properties. The microbiological profiles of each recipe were evaluated for the total count of mesophilic anaerobic bacteria (TMABs), total count of halotolerant bacteria 10 (THBs 10), THBs 17.5, lactic acid bacteria (LABs), fungi, some pathogenic bacteria (*B*. *cereus*, *C*. *perfringens*, and *S*. *aureus*), and *Enterobacteriaceae* on days 2, 4, 6, 8, 18, and 28. All recipes did not contain moderate halophilic bacteria when they were analyzed using procedures THBs 10 and THBs 17.5, respectively. All of the recipes also did not detect fungi, *B*. *cereus*, *C*. *perfringens*, *S*. *aureus*, and Enterobacteriaceae for all observation times. The commercial cincalok from West Kalimantan was reported by Nofiani and Ardiningsih containing THBs 10, THBs 17.5, fungi, and Enterobacteriaceae [7]. The salt added to each recipe probably inhibits most Enterobacteriaceae. Most Enterobacteriaceae cannot grow on high-level salt [24]. Lactic acid produced by LABs also can inhibit the growth of pathogenic and spoilage bacteria (Table 2).

TMABs usually is used to assess the hygienic quality and organoleptic acceptability. In the first six days of fermentation, the TMABs gradually increased even though all of them was around 5 log CFU (Table 3). After six days, the TMABs of recipe A continually increased and began to decrease on day 28. The TMABs of recipe B increased from day 0 to day 3 and fell on day 8, while recipe C tended to be flat (Table 3). After day 8, no TMABs were detected on recipe B. The TMABs of each recipe were higher than those of the commercial cincalok from West Kalimantan (4.66 log CFU) but lower than commercial kecalok from Bangka (5.16 log CFU) and Palembang (5.03 log CFU) [1, 7]. The TA of each recipe on day 8 showed an insignificantly different (*p* > 0.05) but a significant different (*p* < 0.05) pH (Table 2). A low pH value can probably affect the number of microorganisms in this fermentation.

LABs play a role to develop a specific flavour and preservation in fermentation included cincalok. On the 2^nd^-day fermentation of each recipe, LABs around 4 log CFU/g for recipes A and B were detected, while it was around 3 log CFU/g in recipe C (Table 3). The garlic powder probably inhibited the LAB growth on recipe C. However, the number of LABs in each sample fluctuated during fermentation and was not detected after day 18 for recipe A and day 8 for recipes B and C (Table 3). It probably was caused by some LABs in this period was fail to produce the clear zones around colonies that produce a false negative. However, the LAB number of recipes A, B, and C was higher than that of the commercial cincalok from West Kalimantan (1.19 log CFU).

### 3.5. Sensory Analysis of Each Recipe

**Figure 3:**
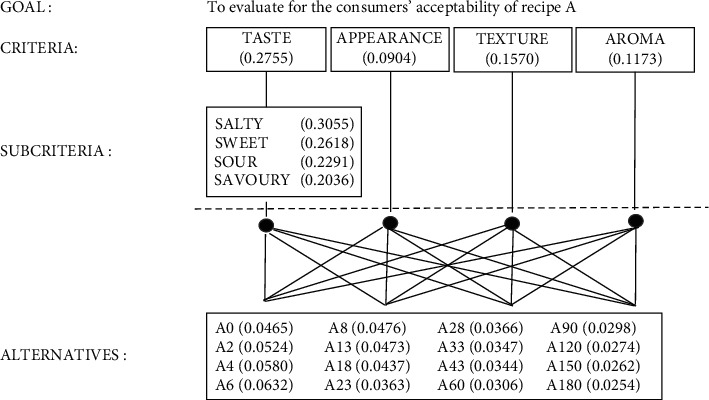
Hierarchy of criteria to evaluate for the consumers' acceptability of recipe A. The values presented the score of each criterion and alternatives. A means recipe A. The number after A means incubation time.

Sensory analysis of each recipe was evaluated using AHP in 2 steps. First, the consumers' acceptability for recipe A was evaluated for 6 months of incubation time on days 0, 2, 4, 6, 8, 13, 18, 23, 28, 33, 43, 60, 90, 120, 150, and 180. The criterion priorities for recipe A from the highest to lowest score based on panellists were taste, texture, aroma, and appearance, respectively (Figure 3). The taste was evaluated using four subcriteria, namely, salty, sweet, sour, and savoury. The panellists gave the highest to lowest subcriterion scores for salty, sweet, sour, and savoury. The panellists showed consistency with their opinion for each criterion which the consistency ratio was less than 10%. Most panellists awarded the highest overall criterion score for recipe A on day 6 of incubation time (Table 4).

**Figure 4:**
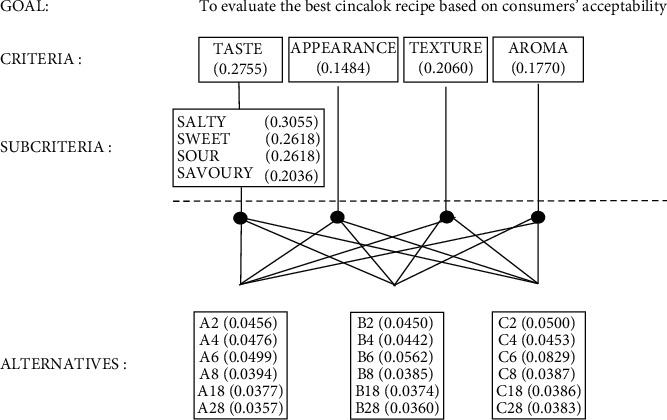
Hierarchy of criteria to evaluate for the consumers' acceptability of each recipe. The values presented the score of each criterion and alternatives: (a). recipe A; (b). recipe B; (c). recipe C. The number after the capital letter means incubation time, day.

**Figure 5:**
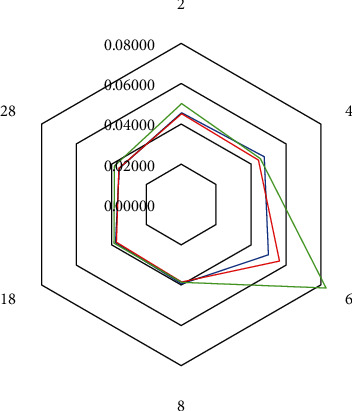
Overall criterion score of each recipe based on AHP analysis. Blue: recipe A; red: recipe B; green: recipe C.

The second sensory evaluation was carried out for recipes A, B, and C on days 2, 4, 6, 8, 13, and 18. The second step aim was to find out the best recipe based on the consumers' acceptability. The order results of the highest to the lowest criteria to evaluate recipes A, B, and C based on the panellists were appearance, aroma, taste, and texture, respectively (Figure 4). All panellists were considered consistent with their choice due to the CR < 10%. The panellists awarded the highest criterion score for all recipes on day 6; however, recipes C gained the highest criterion score, followed by recipes B and A (Table 5, Figure 5).

The sixth day of incubation showed the highest criterion score for all recipes based on the sensory results (Figure 5). The commercial cincalok is usually ripe and ready for sale on days 3-6 of fermentation time with pH product around 5 [7]. Recipes A, B, and C also reached around pH 5 on day 6. The sixth day of fermentation time can be proposed as a cincalok ripeness standard even though each recipe and the commercial cincalok show difference in proximate, physicochemical, and microbiological properties.

## 4. Conclusion

All cincalok recipes showed the best maturity on day 6 based on the sensory analysis. On that day, recipes A, B, and C exhibited similar physicochemical properties, notably the TA, TVBN, AAN, FFA, water, and fat content. Recipes A and B showed insignificant differences (*p* > 0.05) for the pH, crude protein, and ash but were significantly different (*p* < 0.05) for recipe C. All recipes showed differences in the microbiological properties such as the TMABs and LABs. The THBs 10, THBs 17.5, fungi, *B*. *cereus*, *C*. *perfringens*, *S*. *aureus*, and *Enterobacteriaceae* were not detected for all recipes on day 6. However, recipe C (addition of garlic powder) was the most preferred by the consumers then followed with recipes B and C on day 6. The physicochemical, microbiological, and sensory results of cincalok were affected by adding garlic and red chilli pepper powder.

## Figures and Tables

**Table 1:** Proximate compositions of recipes A, B, and C.

Day	Moisture (%)			Ash (%)			Fat (%)			Crude protein (%)		
	A	B	C	A	B	C	A	B	C	A	B	C
0	55.52 ± 0.25^aA^	54.43 ± 0.22^aA^	54.59 ± 0.16^aA^	12.62 ± 0.11^aA^	12.72 ± 0.13^aA^	13.08 ± 0.01^aB^	1.99 ± 2.12^aA^	0.62 ± 0.06^aA^	0.68 ± 0.09^aA^	11.06 ± 1.63^aA^	13.42 ± 1.95^aA^	13.35 ± 1.00^aA^
2	56.25 ± 0.20^aA^	54.79 ± 0.37^aB^	55.31 ± 0.33^adA^	12.51 ± 0.11^aA^	12.48 ± 0.71^aA^	12.90 ± 0.24^aA^	0.53 ± 0.27^aA^	1.65 ± 0.43^aA^	2.98 ± 0.74^aA^	10.66 ± 0.07^aA^	9.75 ± 0.60^aA^	10.40 ± 0.75^acA^
4	49.83 ± 0.63^bA^	52.75 ± 8.20^aA^	47.56 ± 1.37^bA^	12.66 ± 0.04^acA^	12.14 ± 0.55^aA^	13.20 ± 0.42^aA^	1.97 ± 1.90^aA^	0.96 ± 0.38^aA^	1.04 ± 1.31^aA^	10.78 ± 0.57^aA^	10.34 ± 1.19^aA^	9.36 ± 0.14^adA^
6	55.92 ± 0.34^abA^	53.19 ± 3.13^aA^	54.18 ± 0.50^aA^	16.42 ± 0.46^bA^	16.03 ± 0.37^bA^	11.73 ± 0.11^bcB^	1.64 ± 1.11^aA^	2.57 ± 1.02^aA^	1.37 ± 0.23^aA^	10.28 ± 0.01^aA^	11.21 ± 0.44^aA^	9.57 ± 0.32^aB^
8	52.95 ± 2.76^abA^	53.02 ± 1.18^aA^	53.89 ± 1.97^aA^	12.86 ± 0.18^acA^	12.04 ± 0.20^aB^	12.86 ± 0.22^aA^	0.55 ± 0.25^aA^	2.54 ± 2.89^aA^	1.01 ± 0.12^aA^	5.46 ± 0.06^bA^	5.89 ± 0.45^bA^	7.48 ± 0.83^bcdA^
18	54.21 ± 1.29^abA^	56.77 ± 2.51^aA^	58.67 ± 0.91^cdA^	13.15 ± 0.24^adA^	12.73 ± 0.21^aA^	13.26 ± 0.36^aA^	2.02 ± 1.60^aA^	1.95 ± 1.38^aA^	0.84 ± 0.03^aA^	5.30 ± 0.15^cA^	5.15 ± 0.76^bA^	4.63 ± 1.41^bA^
28	60.68 ± 0.46^acA^	60.81 ± 0.04^aAB^	61.80 ± 0.12^cBC^	13.50 ± 0.00^cdA^	12.77 ± 0.26^afB^	12.43 ± 0.04^acB^	1.71 ± 1.02^aA^	0.78 ± 0.04^aA^	1.70 ± 1.68^aA^	5.48 ± 0.62^cA^	6.71 ± .34^bA^	6.48 ± 1.96^bA^

The value is mean ± standard deviation. The lowercase letter in a column indicated a significantly different value (*p* < 0.05). The uppercase letter in a row showed a significant difference (*p* < 0.05). ^A^2000 g of shrimp, 100 g of salt, and 400 g of sugar; ^B^recipe A added 20 g of curly-red chilli pepper; ^C^recipe A added 20 g of garlic powder.

**(a):** 

Day	pH			TA (mM/mL)		
	A	B	C	A	B	C
0	8.20 ± 0.18^aA^	7.94 ± 0.07^aA^	8.12 ± 0.17^aA^	0.35 ± 0.20^aA^	0.25 ± 0.09^aA^	0.25 ± 0.22^aA^
2	7.63 ± 0.18^bA^	7.75 ± 0.07^aA^	7.84 ± 0.01^bA^	0.45 ± 0.01^acA^	0.51 ± 0.15^abA^	0.39 ± 0.13^abA^
4	6.33 ± 0.04^cA^	6.30 ± 0.04^bA^	7.76 ± 0.01^bB^	0.77 ± 0.00^cdA^	0.72 ± 0.13^bcA^	0.78 ± 0.15^bcA^
6	5.46 ± 0.01^dA^	5.49 ± 0.01^cA^	5.93 ± 0.01^cB^	0.88 ± 0.02^bdA^	0.84 ± 0.05^cA^	0.90 ± 0.03^cdA^
8	5.28 ± 0.00^dA^	5.47 ± 0.02^cB^	5.62 ± 0.04^dC^	0.92 ± 0.16^beA^	0.90 ± 0.01^cA^	0.94 ± 0.07^cdA^
18	5.37 ± 0.02^dA^	5.21 ± 0.07^dB^	5.53 ± 0.04^dC^	1.15 ± 0.10^bA^	1.06 ± 0.03^cA^	1.08 ± 0.03^cdA^
28	5.34 ± 0.05^dA^	5.03 ± 0.01^dB^	5.01 ± 0.01^eB^	1.49 ± 0.06^deA^	1.40 ± 0.13^dA^	1.31 ± 0.13^dA^

**(b):** 

Day	TVBN (mg N/100 g)			AAN (mM/g)		
	A	B	C	A	B	C
0	85.51 ± 10.69^aA^	98.95 ± 7.37^aA^	98.28 ± 1.19^aA^	0.65 ± 0.00^aA^	0.64 ± 0.01^aA^	0.67 ± 0.05^aA^
2	102.06 ± 13.66^acA^	107.10 ± 6.53^aA^	97.02 ± 7.72^aA^	0.80 ± 0.05^bcA^	0.71 ± 0.02^abA^	0.69 ± 0.01^acA^
4	121.80 ± 0.00^cdA^	112.14 ± 13.66^bA^	116.76 ± 7.13^bA^	0.72 ± 0.02^aeA^	0.68 ± 0.01^bcA^	0.68 ± 0.06^cA^
6	142.30 ± 0.00^bdA^	159.32 ± 9.82^cA^	139.69 ± 3.68^bA^	0.79 ± 0.02^ceA^	0.73 ± 0.02^bcA^	0.72 ± 0.02^cdA^
8	158.59 ± 5.70^bA^	163.63 ± 12.83^dA^	157.75 ± 6.89^bA^	0.77 ± 0.01^ceA^	0.77 ± 0.02^bcA^	0.74 ± 0.03^cdA^
18	162.54 ± 4.16^bA^	160.02 ± 4.16^bcdA^	160.10 ± 0.24^cA^	0.88 ± 0.02^cA^	0.78 ± 0.05^cdA^	0.84 ± 0.05^cdA^
28	146.32 ± 9.27^bdA^	128.35 ± 17.58^dA^	168.85 ± 10.68^dA^	1.01 ± 0.00^dA^	1.00 ± 0.01^dA^	1.01 ± 0.00^dA^

**(c):** 

Day	FFA (%)		
	A	B	C
0	0.57 ± 0.37^aA^	0.66 ± 0.01^aA^	0.62 ± 0.01^aA^
2	0.65 ± 0.01^aA^	0.74 ± 0.02^bB^	0.66 ± 0.01^aA^
4	0.98 ± 0.01^bA^	1.01 ± 0.00^cA^	0.84 ± 0.01^bB^
6	0.87 ± 0.02^cA^	0.85 ± 0.04^dA^	0.82 ± 0.03^bA^
8	0.98 ± 0.02^bA^	0.93 ± 0.01^eA^	0.81 ± 0.01^bB^
18	0.97 ± 0.03^bA^	0.96 ± 0.02^ceA^	0.94 ± 0.02^cA^
28	1.01 ± 0.00^bA^	1.03 ± 0.01^cA^	1.05 ± 0.01^dA^

The value is mean ± standard deviation. The lowercase letter in a column indicated a significantly different value (*p* < 0.05). The uppercase letter in a row indicated a significantly different value (*p* < 0.05). ^A^2000 g of shrimp, 100 g of salt, and 400 g of sugar; ^B^recipe A added 20 g of curly-red chilli pepper; ^C^recipe A added 20 g of garlic powder.

**Table 3:** TMAB and LABs of recipes A, B, and C.

Incubation time (day)	TMABs (log CFU)			LABs (log CFU)		
	A	B	C	A	B	C
2	5.26	5.53	5.50	4.95	4.36	3.30
4	5.71	5.60	5.49	3.30	-	3.60
6	5.52	5.70	5.87	-	-	3.70
8	6.22	5.07	5.86	4.26	3.78	4
18	6.26	-	5.94	6.05	-	-
28	6.01	-	5.97	-	-	-

-: not detected.

**Table 4:** The score of each criterion for recipe A.

Incubation time (day)	Subcriterion score of taste				Total score of taste	Criterion score			Overall score
	Salty	Sour	Savoury	Sweet		Appearance	Texture	Aroma	
0	0.0867	0.0614	0.0668	0.0518	0.0621	0.0867	0.0744	0.0840	0.0465
2	0.0904	0.0687	0.0688	0.0778	0.0715	0.0904	0.0902	0.0888	0.0524
4	0.0982	0.0693	0.0705	0.1077	0.0875	0.0982	0.0928	0.0892	0.0580
6	0.1053	0.0895	0.0732	0.1196	0.0982	0.1053	0.0999	0.0931	0.0632
8	0.0836	0.0677	0.0639	0.0649	0.0657	0.0836	0.0816	0.0775	0.0476
13	0.0766	0.0661	0.0628	0.0696	0.0660	0.0766	0.0846	0.0758	0.0473
18	0.0656	0.0630	0.0625	0.0569	0.0613	0.0656	0.0799	0.0714	0.0437
23	0.0464	0.0607	0.0618	0.0513	0.0565	0.0464	0.0526	0.0711	0.0363
28	0.0530	0.0442	0.0615	0.0555	0.0575	0.0530	0.0575	0.0593	0.0366
33	0.0493	0.0506	0.0615	0.0550	0.0584	0.0493	0.0481	0.0559	0.0347
43	0.0467	0.0643	0.0613	0.0553	0.0609	0.0467	0.0453	0.0537	0.0344
60	0.0420	0.0604	0.0597	0.0492	0.0575	0.0420	0.0389	0.0414	0.0306
90	0.0413	0.0506	0.0589	0.0500	0.0540	0.0413	0.0435	0.0372	0.0298
120	0.0410	0.0549	0.0573	0.0472	0.0474	0.0410	0.0397	0.0379	0.0274
150	0.0392	0.0627	0.0554	0.0423	0.0465	0.0392	0.0372	0.0338	0.0262
180	0.0348	0.0659	0.0541	0.0460	0.0489	0.0348	0.0339	0.0298	0.0254

**Table 5:** The score of each criterion for recipes A, B, and C.

Incubation time (day)	Subcriterion score of taste				The total score of taste	Criterion score			Overall score
	Salty	Sour	Savoury	Sweet		Appearance	Texture	Aroma	
A2	0.0583	0.0760	0.0352	0.0612	0.0584	0.0562	0.0562	0.0540	0.0456
A4	0.0629	0.0682	0.0846	0.0626	0.0684	0.0565	0.0532	0.0530	0.0476
A6	0.0536	0.0688	0.0339	0.0562	0.0537	0.0641	0.0666	0.0668	0.0499
A8	0.0537	0.0616	0.0325	0.0522	0.0508	0.0485	0.0475	0.0477	0.0394
A18	0.0508	0.0621	0.0339	0.0513	0.0501	0.0455	0.0447	0.0450	0.0377
A28	0.0517	0.0573	0.0316	0.0530	0.0492	0.0422	0.0414	0.0417	0.0357
B2	0.0578	0.0535	0.0341	0.0530	0.0507	0.0600	0.0577	0.0581	0.0450
B4	0.0528	0.0458	0.0305	0.0492	0.0457	0.0613	0.0585	0.0589	0.0442
B6	0.0527	0.0473	0.0345	0.0575	0.0490	0.0656	0.0858	0.0864	0.0562
B8	0.0534	0.0519	0.0298	0.0518	0.0478	0.0489	0.0470	0.0473	0.0385
B18	0.0542	0.0449	0.0368	0.0541	0.0485	0.0464	0.0445	0.0448	0.0374
B28	0.0534	0.0473	0.0338	0.0509	0.0473	0.0425	0.0432	0.0435	0.0360
C2	0.0485	0.0443	0.0286	0.0530	0.0447	0.0728	0.0700	0.0705	0.0500
C4	0.0566	0.0445	0.0457	0.0516	0.0503	0.0607	0.0584	0.0588	0.0453
C6	0.0755	0.1002	0.2763	0.0724	0.1212	0.0934	0.0928	0.0934	0.0829
C8	0.0520	0.0418	0.0542	0.0524	0.0502	0.0479	0.0466	0.0459	0.0387
C18	0.0557	0.0415	0.0597	0.0609	0.0546	0.0454	0.0441	0.0436	0.0386
C28	0.0566	0.0431	0.0846	0.0567	0.0592	0.0422	0.0417	0.0404	0.0383

A: recipe A; B: recipe B; C: recipe C. The number in the incubation time column showed the incubation time.

## Data Availability

The data are presented in the manuscript.
